# Best of both worlds: promise of combining brain stimulation and brain connectome

**DOI:** 10.3389/fnsys.2014.00132

**Published:** 2014-07-30

**Authors:** Caroline Di Bernardi Luft, Ernesto Pereda, Michael J. Banissy, Joydeep Bhattacharya

**Affiliations:** ^1^Department of Psychology, Goldsmiths, University of LondonLondon, UK; ^2^Lab. of Electrical Engineering and Bioengineering, Department of Industrial Engineering, Institute of Biomedical Technology, University of La LagunaTenerife, Spain

**Keywords:** tCS, connectome, graph theory, functional connectivity, structural connectivity, tDCS, tACS, tRNS

## Abstract

Transcranial current brain stimulation (tCS) is becoming increasingly popular as a non-pharmacological non-invasive *neuromodulatory* method that alters cortical excitability by applying weak electrical currents to the scalp via a pair of electrodes. Most applications of this technique have focused on enhancing motor and learning skills, as well as a therapeutic agent in neurological and psychiatric disorders. In these applications, similarly to lesion studies, tCS was used to provide a causal link between a function or behavior and a specific brain region (e.g., primary motor cortex). Nonetheless, complex cognitive functions are known to rely on functionally connected multitude of brain regions with dynamically changing patterns of information flow rather than on isolated areas, which are most commonly targeted in typical tCS experiments. In this review article, we argue in favor of combining tCS method with other neuroimaging techniques (e.g., fMRI, EEG) and by employing state-of-the-art connectivity data analysis techniques (e.g., graph theory) to obtain a deeper understanding of the underlying spatiotemporal dynamics of functional connectivity patterns and cognitive performance. Finally, we discuss the possibilities of using these combined techniques to investigate the neural correlates of human creativity and to enhance creativity.

## Introduction

The possibility of non-invasively modulating the activity of the brain using transcranial current brain stimulation (tCS) has been intriguing the researchers in a variety of fields as it allows to improve cognition in various domains (Fregni et al., 2005; Santiesteban et al., 2012; Schaal et al., 2013; Snowball et al., 2013) or treat many human psychiatric conditions (Boggio et al., 2007, 2008; Rigonatti et al., 2008; Nitsche et al., 2009; Terhune and Cohen Kadosh, 2013). There are a number of tCS techniques available, including, but not limited to, transcranial direct current stimulation (tDCS), transcranial alternating current stimulation (tACS), and transcranial random noise stimulation (tRNS) (for a review on the tCS methods, see: Nitsche et al., 2008; Ruffini et al., 2013). In tDCS, a small direct current (DC) is passed from anodal (positive) to cathodal (negative) electrodes positioned in the head surface in order to target specific brain areas underneath the electrodes (Nitsche and Paulus, 2000; Faria et al., 2011). Early studies with animals demonstrated an increase in excitation through membrane depolarization in the neurons underneath anodal electrode but an inhibition under the cathodal one (Bindman et al., 1962, 1964; Purpura and McMurtry, 1965). In humans, there is evidence for an increase in excitability in areas underneath the anodal electrode and a decrease underneath the cathodal following tDCS on the motor (Nitsche and Paulus, 2000) and visual cortex (Antal et al., 2004). Although this rationale of higher excitability under anodal and inhibition under cathodal has been used for determining the stimulation protocol in many studies, it remains unclear if this is so in all cases, as other variables such as the position of the cathodal in relation to anodal (Nitsche and Paulus, 2000; Antal et al., 2004; Moliadze et al., 2010) and the intensity of the stimulation (Batsikadze et al., 2013) seem to interfere with the excitability effects observed under anodal and cathodal stimulation sites. In tRNS the areas underneath both electrodes are stimulated with a current whose amplitude varies randomly in time within the frequency range of 100–640 Hz (Terney et al., 2008; Ruffini et al., 2013). In tACS, an alternating current (AC) with a pre-determined frequency passes from anodal to cathodal and the frequency is usually set within the EEG frequency spectrum (1–100 Hz) (Antal et al., 2008; Kanai et al., 2010).

The protocol for tCS stimulation, especially the anodal and cathodal electrodes location, is usually determined based on neuroimaging findings (e.g., EEG, fMRI) evidencing that a certain region is involved in the target brain function which the researcher wants to modulate. Therefore, most tCS studies hitherto are grounded on the modular paradigm, in which complex cognitive functions are thought to be mediated by independent brain areas (e.g., Kanwisher et al., 1997). Despite the great advance in the knowledge made through the modular paradigm in the last decades, the understanding that each cognitive function is mediated by independent brain areas is challenged by an increasing number of studies supporting that most cognitive functions are mediated by widely distributed areas functioning in parallel (Fuster, 2000; Sporns, 2014). For example, dyslexia was for a long time thought to be caused by a problem in the phonetic representations located in the primary and secondary auditory cortices (Goswami, 2000). However, recent work (Boets et al., 2013) has shown that dyslexic individuals have intact phonetic representations, but presented a problem in connectivity, both structural and functional, between inferior frontal gyrus (IFG) and the bilateral auditory cortex, which is associated with retrieving these representations. Other disorders such as schizophrenia (van den Heuvel et al., 2013), epilepsy (Bettus et al., 2008), and autism (Barttfeld et al., 2011) are also associated with abnormal (increased or decreased) brain connectivity rather than abnormal activity of isolated brain regions. In such cases, in seems logical that brain stimulation should not target one or the other isolated area, but the connection between them, which is certainly a challenging aim because most of the brain stimulation effects are assumed to be caused by the excitation/inhibition of the specific areas underneath anodal/cathodal electrodes.

Thus, in order to target specific connections rather than specific areas, it is necessary to understand how (or even whether) brain networks respond under or after tCS. In fact, the notion that tCS effects are brought about by increases/decreases in activation of the stimulated area has been challenged by studies showing that the effects of tDCS are not restricted to the stimulated sites (Lang et al., 2005; Kwon et al., 2008; Keeser et al., 2011). Moreover, there are some recent studies showing that tDCS affects brain connectivity patterns during both task and rest (Keeser et al., 2011; Polania et al., 2011a,b, 2012a; Meinzer et al., 2012, 2013), suggesting that the tCS has an impact not only on the target areas, but also on the brain networks. In this review paper, we discuss the possibility of tracking tCS-induced changes in the brain network by combining neuroimaging with advanced connectivity analysis techniques (e.g., graph theory). We briefly review the mechanisms of tCS and the basics of brain network analysis through graph theory as a framework to develop new brain stimulation protocols able to produce relevant changes in brain connectivity and, ultimately, in the features of a given brain network. In particular, we discuss the rationale for determining the stimulation protocol for improving creativity as an example of a complex cognitive construct which requires complex associations between multiple brain areas.

## Possible mechanisms of tCS

Before discussing the macro effects of tCS on connectivity, a brief discussion of its potential mechanisms is needed. Dissertating in detail on how the distinct tCS techniques can affect brain and behavior is out of the scope of this paper, but some of the key issues are elucidated here as they may be important for understanding how tCS can shape brain functional connectivity. Previous research with animals (Bindman et al., 1962, 1964; Purpura and McMurtry, 1965) showed that a small electrical DC passing through the anodal electrode can depolarize the cell membrane at subthreshold intensity, making the neurons more susceptible to excitatory activity as they become less negative, whereas the current passing the cathodal electrode polarizes the cell membrane making it more negative, inhibiting neural firing. Gartside (1968) found that a weak electrical current induced an increase in cortical firing under the stimulated area (anodal) in rats. Importantly, the same study observed that turning off the electrical current after 5 min of stimulation did not cease this increased neural discharge, termed the “aftereffect.” Although in humans the currents reaching the brain through tCS are much weaker than the ones in animal studies (even if the external stimulating current is the same as humans have thicker skull), there is evidence that the brain areas underneath the anodal electrode are more prone to excitatory activity (Nitsche and Paulus, 2000, 2001; Antal et al., 2004). Repeated firing, or high-frequency stimulation, may result in long-term potentiation (LTP) and long-term depression (LTD) (Bliss and Lomo, 1973), which are thought to be the main mechanisms by which tCS modulates brain activity, as it does during learning (Rioult-Pedotti et al., 2000). In the LTP process, sustained activation of the cell through the binding of glutamate to α-Amino-3-hydroxy-5-methyl-4-isoxazolepropionic acid (AMPA) receptors in the post-synaptic membrane causes the magnesium (Mg^2+^) to leave the N-methyl-D-aspartate receptor (NMDA) ion channel, allowing large quantities of calcium (Ca^2+^) to enter the cell through this channel (Mg^2+^ blocks the NMDA channel). These large quantities of Ca^2+^ in the post-synaptic dendrites can improve the synaptic efficiency for an extended time period by activating second messengers (calcium-dependent kinases such as the Ca^2+^/calmodulin-dependent ones: CaMKs), which create more AMPA receptors and protein expressions (growth factor), thereby facilitating neural plasticity (Malenka and Bear, 2004). The role of LTP on tCS effects is supported by pharmacological studies showing that the administration of an antagonist of the NMDA receptor blocks the effects of anodal and cathodal stimulation on the motor evoked potential (MEP), as triggered by a transcranial magnetic stimulation (TMS) pulse, an indirect measure of motor cortex excitability (Liebetanz et al., 2002; Nitsche et al., 2003; Monte-Silva et al., 2013). Saturation of the LTP can induce LTD (Rioult-Pedotti et al., 2000), which might be one of the reasons why the tCS effects were found to be dosage dependant (Batsikadze et al., 2013). It is important to notice that the electrical currents delivered by tDCS/tACS and tRNS are not strong enough to fire an action potential (Radman et al., 2009), but they can cause a bimodal polarization effect, namely soma depolarization and apical dendrite hyperpolarization (Bikson et al., 2004). Therefore tCS, as opposed to TMS (Terao and Ugawa, 2002), affects the post-synaptic potential by promoting a change in the cell gain (Rahman et al., 2013), and not by increasing the firing itself. Furthermore, single neuron response to weak current stimulation seems to be rather dependant on the network dynamics. It was found that low frequency AC currents can induce changes in gamma oscillations (25–35 Hz) in a zero-sum fashion as increases in excitability were balanced by complimentary inhibitory activity according to the network dynamics (Reato et al., 2010). Other studies (Parra and Bikson, 2004; Deans et al., 2007) also revealed that the network plays a role even at the cellular level (*in vitro* studies), as the neuronal firing behavior was largely determined by the network it belongs to.

The ongoing neuronal oscillatory activity during stimulation seems to shape the effects of tDCS and tACS on the resulting network activity (Frohlich and McCormick, 2010; Ali et al., 2013; Frohlich and Schmidt, 2013). This appears to happen through a feedback loop between the neural activity and the endogenous electric field (Frohlich and McCormick, 2010). Thus, tACS at the endogenous oscillation frequency (*in vitro*) produced a higher enhancement of this oscillation as compared to tDCS (Ali et al., 2013). In addition, these authors found that the network oscillatory effects were more pronounced if the stimulated frequency matches the endogenous oscillation frequency, suggesting a resonance-like effect. The importance of network activity was not only evidenced at the cellular level, but also at the cortical level in awake human beings. In relation to tDCS, it was found that stimulating over the premotor cortex (PMC) resulted in increased excitability (as measured by the MEP) over the primary motor cortex (M1) (Boros et al., 2008), which suggests that stimulating one area can affect others structurally connected to it. This might explain why many studies found that the stimulation with weak currents affects a number of areas other than the region underneath the anodal electrode (Lang et al., 2005; Kwon et al., 2008; Keeser et al., 2011). The possibility that network dynamics also plays a role in the effects of tCS at the macro level is supported by studies showing that tDCS brings about changes in functional connectivity, especially when those changes are assessed during task performance (Polania et al., 2012a; Weber et al., 2014). The default mode network (DMN) (Keeser et al., 2011; Amadi et al., 2014) and the attention network (AN) (Pena-Gomez et al., 2012) also seem to be affected by brain stimulation, even when the stimulated area is not within the same network. However, studies investigating the effects of tCS on brain connectivity during different tasks are still scarce (Polania et al., 2011b, 2012a,b; Meinzer et al., 2012, 2013; Weber et al., 2014), but they may shed new light into how tCS affects the brain dynamics and behavior. One of the challenges on this enterprise is to develop a suitable framework that can guide not only the analysis and interpretation of complex connectivity results derived from various neuroimaging techniques, but also to develop more efficient protocols to tackle connectivity.

## Network analysis and brain stimulation

There are many ways of measuring how different brain areas interact or communicate with each other. Connectivity is usually investigated from three perspectives: structural, functional and effective (Friston, 2011). Structural connectivity refers to the anatomical connections within the brain, such as axons and synapses, that can be measured non-invasively through diffusion tensor imaging (DTI) (van den Heuvel and Sporns, 2013). The brain structure is thought to shape or determine the paths for the communication between brain areas while the brain is engaged in various tasks or even at rest (Friston, 2011). While structural connectivity traces the paths between regions based on the physical connections between them, functional and effective connectivity estimate these connections based on the relationship between the time series from each of these brain regions (voxels) or the corresponding electrode / sensor (van den Heuvel and Sporns, 2013). Functional connectivity refers to how interdependent the activity between two areas (or more) is, with no information on the direction of their communication, whereas effective connectivity refers to the directed (source and sink) connection between two areas (Horwitz, 2003; Friston, 2011). Both functional and effective connectivity are dynamic and can be measured from data collected using various neuroimaging techniques, such as EEG, MEG, and fMRI. A variety of algorithms has been developed to estimate functional (e.g., coherence, phase synchronization, correlation, synchronization likelihood), and effective (e.g., dynamic causal modeling, Granger causality, phase slope index) connectivity, and they are available in many neuroimaging analysis toolboxes (e.g., Delorme and Makeig, 2004; Oostenveld et al., 2011; Niso et al., 2013). These measures allow us to estimate the strength of the communication between regions/sensors and they all have different advantages and limitations (Pereda et al., 2005; Friston, 2011).

**Figure 1:**
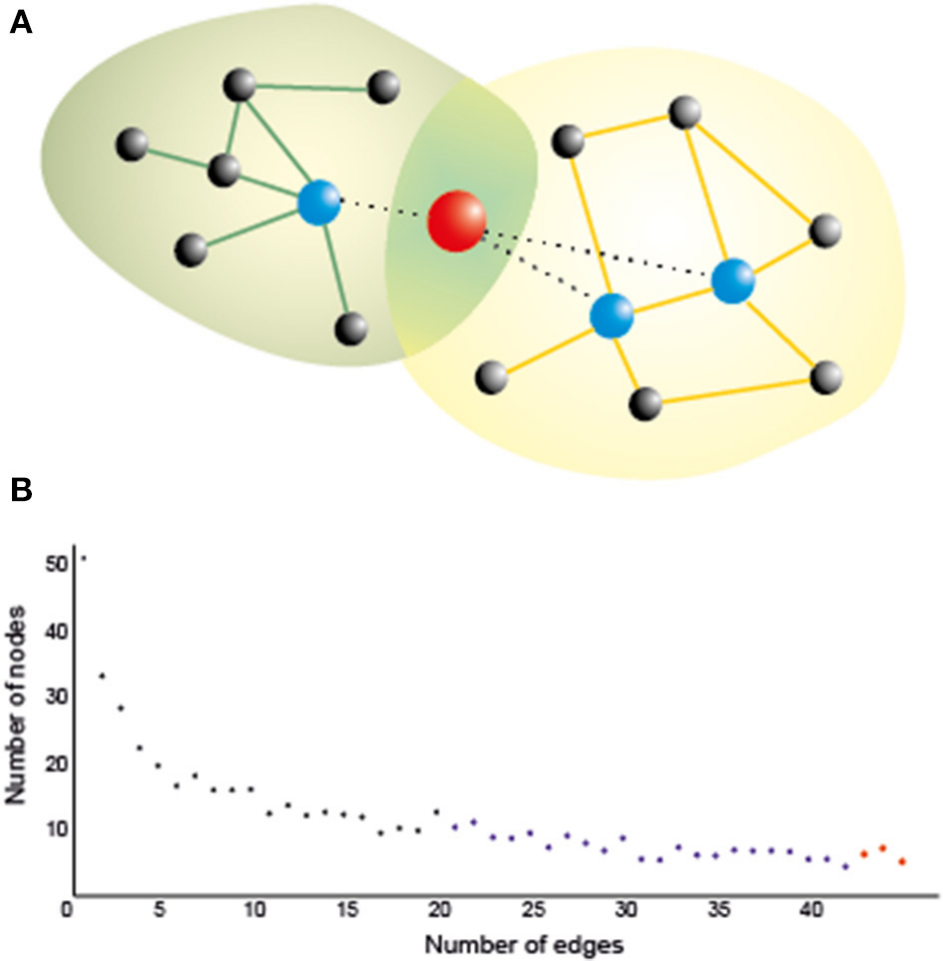
**(A)** A hub naturally serves as a bridge between two or more networks, as it is connected to many nodes in all of them, thereby playing an important role in brain functioning. Stimulating a hub is likely to affect many nodes even at a long distance, which could maximize the effect of stimulation. Yet networks are also vulnerable against a directed attack on the hubs, so that their inhibition, however partial and/or transient, may crucially affect the corresponding cognitive functions if it is not properly controlled (e.g., by multisite stimulation of nearby nodes). *Green and orange lines*: intra-network edges. *Dotted lines*: edges connecting the hub with nodes in both networks; **(B)** A typical degree distribution for brain nodes as assessed by a functional connectivity index. Most of the nodes (black) have low degree (i.e., are connected only to a few nodes); some of them (blue) have moderate degree, and a few of them (in red) are hubs, which are connected to many nodes.

In order to understand the organizing principles of the brain networks (estimated using the techniques mentioned in the previous paragraph), we can use graph theory, which has emerged as an important model for understanding and quantifying the global properties of brain networks (Bassett and Bullmore, 2006; Bressler and Menon, 2010; Sporns, 2011). In graph theory, networks are mathematically represented as a set of *nodes*, which in this framework are the brain regions or the electrodes/sensors for EEG/MEG, connected through *edges*, which are the paths or lines representing the direct relation between the nodes (Figure 1A). Out of the many measures that can be used to characterize these networks (Rubinov and Sporns, 2010; Sporns, 2014), two of them are allegedly the most commonly used in neuroscience: (1) the average clustering coefficient, and (2) the average shortest path length. The clustering coefficient is the probability that neighboring nodes will be connected. The average shortest path length is the average minimum number of edges or connections that need to be traversed between two nodes. The arrangement of the edges in the network can take three main forms, which in turn determines the character of the network itself: regular, random, and small-world (Watts and Strogatz, 1998). In a regular network, each node is connected to its neighbors, resulting in high clustering and large average path length. If we randomly rewire most of the edges in a regular network, we would reduce the path length and the clustering coefficient, which characterizes a random network. However, if only a small number of edges are rewired to connect distant nodes, we have a “small-world” network, characterized by a small shortest path length and a high clustering coefficient (Watts and Strogatz, 1998). In small-world networks, the nodes containing the long-range connections have a similar number of connections in comparison to the other nodes, which makes the network resistant to random attack to the nodes. In the brain, however, there are some regions (termed network hubs) that are more heavily connected than others, and also more connected among themselves (van den Heuvel and Sporns, 2013). This type of network organization, with only a few nodes more connected than others and also more connected among themselves, is known as “scale-free,” because such networks have a power law degree distribution (Barabasi and Albert, 1999; Sporns et al., 2004), which means that most nodes have only few connections or edges, whereas a few hubs have a large number of connections, as represented in Figure 1B. The presence of hubs (blue and red nodes in Figure 1), which are heavily connected and usually also centrally located nodes, and associated with locally connected specialized nodes, allows the network to have both local and global information processing (van den Heuvel and Sporns, 2011, 2013). The hubs are essential for brain communication, albeit energetically expensive as they are highly connected, and occupy a privileged position in the network, connecting distant communities of nodes to other hubs. In addition these hubs are organized as a “rich-club,” in which densely connected nodes tend to be more connected to each other (van den Heuvel and Sporns, 2013). There are two types of rich-club hubs: connector hubs (red node Figure 1A), which interconnect different modules, and provincial hubs (blue nodes Figure 1A), which connect nodes within the same module. A study with DTI (van den Heuvel and Sporns, 2011) identified three main cortical areas (both hemispheres, near the midline) which are connector hubs: superior parietal, precuneus, and superior frontal cortex; and three subcortical regions (both hemispheres near medial regions) which are provincial hubs: putamen, hippocampus, and thalamus. Importantly, they found that attacking the rich-club connections (links between members of the rich-club) in comparison to random attacks or attacks to other hub connections caused a larger decrease in the global efficiency of the network. These hubs were defined based on structural connectivity, but there is accumulating evidence on the large overlap between brain network structure and functional connectivity especially during resting state (Cabral et al., 2014; Goni et al., 2014). Regions within the DMN, medially, contain most of the functional hubs, including most of the medial regions, including anterior cingulate cortex (ACC), precuneus/posterior cingulate gyrus. Using a measure of global functional connectivity, a study (Cole et al., 2010) found that not only the DMN regions have high functional connectivity with all other regions, but the cognitive control network (CCN) (Cole and Schneider, 2007), which comprises the dorsolateral prefrontal cortex (DLPFC), rostrolateral prefrontal cortex (RLPFC), dorsal-caudal ACC, inferior frontal junction (IFJ), posterior parietal cortex (PPC), PMC, and anterior insular cortex (AIC), has also high global connectivity. The regions in the DMN and in the CCN are among the top 5% most connected regions in the brain (Cole et al., 2010). Recently, the concept of flexible hubs (Cole et al., 2013a,b), which are brain regions (e.g., DLPFC) that quickly shift their functional connectivity patterns (become highly connected) to implement cognitive control, has been discussed as an important part of network's ability for flexible behavior. Besides, a recent theoretical work (Aguirre et al., 2014) shows that communication through heavily connected nodes facilitates synchronization between different networks. Therefore, the structural and functional hubs seem to be an essential part of the scale-free brain network organization.

Making use of its structural and functional hubs, which can flexibly adapt to different environmental demands, the scale-free configuration allows dynamical exchange of information that facilitates parallel processing and rapid changes on its own configuration (Bassett and Bullmore, 2006). There is evidence that the scale-free network characteristics, as measured by graph theory, are optimized during awake compared to sleep periods (Uehara et al., 2014), suggesting that the functional organization of the network is relevant for cognitive processing. Therefore, graph-theory can be used to investigate structural, functional and effective connectivity. Functional connectivity gives undirected edges to the network, whereas effective connectivity provides information on the direction of the interactions investigated. Moreover, the edges can be weighted according to the degree of coupling between two nodes, as estimated by the techniques mentioned previously. Knowing and estimating the changes in network properties as a result of brain stimulation, during task and resting state alike, has important implications on the understanding of its effects over behavior. In addition, the knowledge of the networks representing the target cognitive process might provide insight into optimizing stimulation protocols.

## Brain stimulation and connectivity

There is a great deal of research analysing the impact of TMS on brain connectivity patterns during task and rest (for a review, see Shafi et al., 2012). As TMS is not covered in this paper, we only summarize four main findings on TMS-induced alterations in connectivity, which can shed light onto how connectivity changes in response to brain stimulation: (1) *state dependency:* the brain state during stimulation affects how it modifies connectivity (Massimini et al., 2005; Davare et al., 2008; Morishima et al., 2009); (2) *rich-club spreading:* stimulating areas that have more connections (rich-club nodes) will affect a larger network (Bestmann et al., 2003, 2005; Chouinard et al., 2003); (3) *structural connectivity spreading:* stimulating one area affects other regions that are structurally connected to the main stimulated region (Pascual-Leone and Walsh, 2001; Mochizuki et al., 2004); (4) *compensatory connectivity:* inhibiting certain brain areas may trigger compensatory activity in the task related network (O'Shea et al., 2007). Although there are not many studies on the effects of tCS on brain connectivity, we will discuss some of these premises in the context of the few available ones published hitherto, which are listed in Table 1.

**Table 1:** **Studies on the effects of tCS on brain connectivity**.

**Study**	**Technique**	**Parameters/location**	**Duration**	**Study design**	**Results**
Polania et al., 2011a	tDCS	1 mA 4 × 4 cm^2^ Anode: left M1 Cathode: right frontopolar	10 min during rest	Within participants (two sessions) Conditions: (1) tDCS; (2) Sham	After tDCS, not sham, there was an increase in synchronization between the stimulated area with premotor and sensorimotor more pronounced in the gamma band and during the motor task
Polania et al., 2012a	tDCS	1 mA 7 × 5cm^2^ Anode: left M1 Cathode: right frontopolar	10 min during rest	Within participants Conditions: (1) Anode left M1/cathode right frontopolar; (2) Anode right M1/cathode left frontopolar; (3) Sham	Anode over left M1 was associated with increased connectivity between left thalamus and the ipsilateral M1, and between left caudate and parietal association cortex. Connectivity between the caudate and regions of the default mode network (DMN) was reduced, especially with the PCC. Cathode over the left M1 resulted reduced connectivity: between right putamen and left precentral gyrus and between right thalamus and left superior frontal gyrus
Meinzer et al., 2012	tDCS	1 mA Anode: 5 × 7 cm^2^ Cathode: 10 × 10 cm^2^ Anode: left IFG (BA44/45) Cathode: right supraorbital	20 min of which: ~6 min during rest ~11 min during task	Within participants Conditions: (1) tDCS; (2) Sham	Anodal tDCS over the left IFG was associated with better performance in a semantic word generation task. The BOLD response reduced at stimulated areas (left ventral IFG) compared to sham during the task. An increase in connectivity in the language network areas (left IFG and anterior insula) was also observed during the tDCS compared to sham. During rest, anodal stimulation resulted in increased connectivity between the left ventral IFG and other major language network hubs which partially overlapped with the task related changes in connectivity
Meinzer et al., 2013	tDCS	1 mA Anode: 5 × 7 cm^2^ Cathode: 10 × 10 cm^2^ Anode: left IFG (BA44/45) Cathode: right supraorbital	20 min of which: ~6 min during rest ~11 min during task	Within participants Conditions: (1) tDCS; (2) Sham	Older participants under sham stimulation performed a semantic word generation task worse than the younger group. However, during the tDCS, there was no difference in the performance between old and young participants. During task, the stimulation was associated with lower BOLD response at the stimulated site (left IFG—anodal), but with increased connectivity between this and other areas of the language network. The differences in BOLD and connectivity between young and older were reduced during tDCS stimulation, evidencing a “youth-like” effect in the older participants' brains under stimulation
Keeser et al., 2011	tDCS	2 mA 7 × 5 cm^2^ Anode: left DLPFC Cathode: right supraorbital	20 min during rest	Within participants Conditions: (1) tDCS; (2) Sham	Among four resting state networks: DMN, left and right frontal-parietal networks (FPNs) and the self-referential network, it was found that tDCS induced a change in connectivity within the DMN and the FPNs
Pena-Gomez et al., 2012	tDCS	2 mA 7 × 5 cm^2^ Anode: DLPFC Cathode: right supraorbital	20 min during rest	Within participants Conditions: (1) Anodal left DLPFC; (2) Anodal right DLPFC; (3) Sham	Anodal tDCS to the DLPFC resulted in an increase in functional connectivity between prefrontal and parietal regions. There was also a decrease in the spatial configuration of the DMN following both right and left DLPFC anodal stimulation
Sehm et al., 2013	tDCS	1 mA Unilateral: Anode: right M1 Cathode: contralateral orbit; Bilateral: Anode: right M1 Cathode: left M1	20 min during rest	Within participants Conditions: (1) Unilateral; (2) Bilateral; (3) Sham	Bilateral tDCS was associated with reduced interhemispheric connectivity during stimulation and with an increase in intracortical connectivity within right M1 after the stimulation. Unilateral tDCS was associated with reduced interhemispheric connectivity, but not with increased connectivity after the stimulation as did the bilateral
Alon et al., 2011	tDCS, tPCS	tDCS: 2 mA 7 × 4.5 cm^2^ Anode: right M1 Cathode: left supra-orbital tPCS: Monophasic waveform with pulse duration of 33 us and interval of 33.3 us Stimulator's carrier frequency 15 kHz	12 min 48 s (split in two—STIM 1 and STIM 2 with 6 min 24 s each)	Within participants Conditions: (1) tDCS; (2) tPCS	A reduced resting functional connectivity between right and left M1 was found after stimulation in both tDCS and tPCS
Weber et al., 2014	tDCS	1.5mA 5 × 5 cm^2^ Anode: right DLPFC (F4) Cathode: left DLPFC (F3)	15 min rest outside scanner	Between subjects: Groups: (1) tDCS; (2) Sham	The tDCS group showed reduced connectivity between the right ACC and the rest of the brain after the stimulation during rest
Chib et al., 2013	tDCS	2 mA Anode: 3.5 × 3.5 cm^2^ VMPFC (Fpz) Cathode: 5 × 5 cm^2^ Right DLPFC (F4)	15 min during rest	Between subjects Groups^*^: (1) Main Stimulation; (2) Active sham group: Anode 5 × 5 cm over right DLPFC; and Cathode 3.5 × 3.5 cm over VMPFC Within-subjects: pre- vs. post-stimulation	Functional connectivity changes elicited by tDCS were evaluated during a face attractiveness judgment task, before and after stimulation. The main stimulation was associated with an increase in connectivity between the VMPFC and the midbrain area (substantia nigra and ventral tegmental area). The higher the functional connectivity between these two areas during stimulation, the better the performance in the face judgment task
Neuling et al., 2013	tACS	Individualized current around 1500 mA 5 × 7 cm^2^ Anode: Oz Cathode: Cz Stimulation frequency: individual alpha peak frequency (IAF)	20 min during an auditory detection task	Between subjects Experiment eyes-closed groups: (1) tACS; (2) Sham Experiment eyes-open groups: (1)tACS; (2) Sham	They found that the aftereffects of tACS were higher for the group whose stimulation was done with eyes-open, whereas it did not differ between stimulation and sham for the eyes-closed experiment. However, the coherence between right and left parietal (P4-P3) increased only for the tACS group of the eyes-closed experiment

* *Other four conditions were tested behaviorally, but only the main stimulation was effective in improving attractiveness judgments, leading them to scan only this and the active sham group for comparison*.

Most of the papers investigating the effect of tCS on brain connectivity analyzed the changes in the functional network during resting state (Alon et al., 2011; Keeser et al., 2011; Polania et al., 2011b, 2012a; Meinzer et al., 2012, 2013; Pena-Gomez et al., 2012). Studies vary in how they define the regions/nodes of the network. Using fMRI, some studies looked into the connectivity using M1 as a seed (Alon et al., 2011; Polania et al., 2012a; Sehm et al., 2013), while others looked into specific regions of interest (Pena-Gomez et al., 2012; Polania et al., 2012b; Chib et al., 2013; Weber et al., 2014), or into the resting state networks (Keeser et al., 2011; Pena-Gomez et al., 2012). Furthermore, graph-theory was also used for tracking connectivity changes after brain stimulation (Polania et al., 2011a).

The results on stimulating motor cortex (M1) are somehow mixed: some found increased functional connectivity within M1 (Polania et al., 2012a), whereas others found a decrease (Alon et al., 2011) or both a decrease during the stimulation but an increase afterwards (Sehm et al., 2013). One of the issues with these studies is that functional connectivity before and after stimulation was assessed during rest, yet M1 is not typically a region which is highly active during rest (Boros et al., 2008), so the impact of stimulation on brain connectivity may not be pronounced or strong as the motor network is relatively idle during rest. This possibility is supported by an EEG study on the effects of tDCS over M1 on functional connectivity during rest and during a finger tapping task (Polania et al., 2011a). These authors found that stimulation of the left motor cortex (anodal over C3/C5) with the cathodal electrode over right frontopolar electrodes (Fp2) was associated with higher connectivity of the motor areas in the gamma frequency band (60–90 Hz) during finger tapping. In addition, they found an increase in frontal connectivity in theta (4–7 Hz) and alpha (8–12 Hz) frequency bands during rest after tDCS stimulation, but this result was weaker than during task. They also found reduced coupling between frontal and occipital and areas after tDCS as compared to sham stimulation, which indicates that brain stimulation can shape connectivity not only by increasing communication between areas directly associated with the performing task, but also by reducing communication between other areas. Altogether, these results showed that the findings 1 (state-dependency) and 4 (compensatory connectivity) mentioned above can also explain some of the changes in functional connectivity following tDCS, while the aftereffects of the stimulation are still in place. The state-dependency in the cited studies is not related to the exact activity during the stimulation itself as referred in the TMS studies (Massimini et al., 2005; Davare et al., 2008; Morishima et al., 2009), but with the task conducted during the aftereffects of the stimulation. This means that the increase in coupling resulting from the stimulation is dependent on the task performed during the aftereffect period and on whether it recruits the stimulated network. In relation to finding 4 (compensatory connectivity), it seems that connectivity also changes in a zero-sum fashion, as it was suggested to be the case for most neuroenhancement interventions (Brem et al., 2014).

The state dependency seems to be important not only for tDCS, but also for tACS. By investigating how it can boost motion discrimination and lower adaptation, a study found that the method was only effective when the 10 Hz stimulation over the motion area (left hMT+) was administered during visual stimulation, but not before or after it (Kar and Krekelberg, 2014). This suggests that, differently from the tDCS, where the task conducted during the aftereffects can shape the effects of the stimulation, tACS effects on brain synchronization are dependent of the ongoing activity/task or brain state during which the stimulation is administered. In the latter study (Kar and Krekelberg, 2014), administering tACS during rest is unlikely to boost motion discrimination since the effects seem to be very dependent on the precise moment of visual perception in each trial.

The structural connections between brain areas, as in findings 2 (rich-club spreading) and 3 (structural connectivity spreading), also seem to be of importance for understanding how tCS affects the brain networks. It is possible to use the knowledge about the connectivity between areas to target deeper brain structures (Takano et al., 2011; Chib et al., 2013) which up until recently could only be targeted pharmacologically. Research with animals (Takano et al., 2011) observed an increased activation in the nucleus accumbens in rats after 10 min of stimulation over the frontal cortex. A recent study in humans (Chib et al., 2013) used tDCS to target midbrain areas (subcortical), including substantia nigra (SN) and the ventral tegmental area (VTA) by stimulating the ventral medial prefrontal cortex (VMPFC), during a face attractiveness judgment task, which is associated with the dopaminergic system for reward processing. The anodal and cathodal electrode locations were defined based on the knowledge that excitation of the VMPFC combined with inhibition of the DLPFC can bring about an increase in activity (together with dopamine release) of the midbrain (Takano et al., 2011). They found that the main stimulation groups (anodal over the VMPFC and cathodal over the DLPFC) increased their ratings of face attractiveness after the stimulation, but not the active sham group, which was stimulated for the same time, but with the opposite locations (anode over DLPFC and cathode over VMPFC). Importantly, the main stimulation elicited an increase in the BOLD signal in the midbrain areas and an increase in functional connectivity between VMPFC and the midbrain area. This increase in connectivity was correlated to the participants' ratings of attractiveness. In addition, the behavioral effect was only evident when the cathodal electrode was placed over the DLPFC, as the same effects were not found when the cathode was positioned on the vertex (Cz). This result is consistent with the idea that coordinated activity between different brain areas, in this case VMPFC and DLPFC, can influence the outcomes of the stimulation in subcortical areas. Relevantly, it also demonstrates that it is possible to exploit the brain networks structure to target subcortical areas and their connections. The ability to affect connections between cortical and subcortical areas by stimulating the cerebral cortex has been also demonstrated during rest (Polania et al., 2012a).

Studies looking at resting state networks (Keeser et al., 2011; Pena-Gomez et al., 2012) found changes in functional connectivity after stimulation of the DLPFC. One of these studies (Pena-Gomez et al., 2012) found that stimulating either the right or the left DLPFC resulted in robust changes in the AN and in the DMN. It was observed that anodal tDCS over DLPFC was associated with a disruption in the DMN topography, as if the anterior (medial prefrontal) and posterior components (medial posterior) of the network become temporally independent after the stimulation. On the other hand, there was an increase in functional connectivity between frontal and parietal areas, which are part of the AN. An increase in fronto-parietal connectivity related to the AN was also found by others (Keeser et al., 2011), along with an increase in connectivity between an area near the anodal electrode on the left DLPFC and the DMN regions. It may be that tDCS over the DLPFC increases the alertness for action as indicated by the networks affected by stimulation, which it was suggested by the authors of both studies (Keeser et al., 2011; Pena-Gomez et al., 2012). As we mentioned in the previous section, the DLPFC can be considered a functional “flexible hub,” which plays an important role in switching from one state to the other in order to attend to the necessary task demands (Cole et al., 2013b). This might be one of the reasons why stimulating DLPFC seems to affect functional connectivity in the DMN, as it reflects the change from one to the other network. These studies support that tCS can alter the connectivity in the brain during rest. In order to improve the interpretation of the results, new studies should target specific networks and bring new hypothesis of possible behavioral correlates. For example, what does it mean to increase or decrease connectivity in certain brain networks? Would these changes, for instance, improve mood, reduce depression, increase alertness, and others? The need for behavioral correlates in these studies is crucial for understanding their functional meaning.

Hitherto, two recent studies (Meinzer et al., 2012, 2013) used fMRI to monitor the brain activity during rest as well as during a semantic word generation task while the participants received anodal tDCS over the left IFG (targeting language areas—BA 44/45—see Table 1 for details). Both studies observed a reduction in the BOLD signal at the stimulated sites during the language task. Importantly, the connectivity between the stimulated area (left IFG) and other language-related area was increased during stimulation for both task and rest (Meinzer et al., 2012, 2013). It has been argued that these changes are related to increased neural efficiency at the stimulated site and its networks (Kar and Wright, 2014) similar to the changes observed as a result of learning (Buchel et al., 1999). These changes were found to be behaviorally relevant as the performance in the semantic word generation task improved when the participants were receiving active stimulation compared to sham. One of these studies (Meinzer et al., 2013) observed an interesting effect whereby older adults showed a pattern similar to that of their younger counterparts in terms of performance in the semantic task when receiving anodal stimulation over the left IFG, despite performing worse without stimulation. In a similar vein, the differences in BOLD activation and functional connectivity between young and older adults were reduced during anodal tDCS. In relation to the connectivity, older participants showed higher anterior (fronto-temporal, and medial frontal regions) and lower posterior (temporo-occipital, precentral, and postcentral cortices) functional connectivity than younger, which was related to worse task performance. Anodal tDCS stimulation over the left IFG seems to reverse these effects, as it reduced functional connectivity among anterior cortical areas and increased it among the posterior cortices (note that not all the age related connectivity differences were reversed though).

Considering that all but one of these studies (except Polania et al., 2011a), were conducted using tDCS in combination with fMRI, more research is needed using other tCS techniques, especially tACS and tRNS, combined with higher temporal resolution neuroimaging techniques such as EEG/MEG, as we cannot expect that all tCS techniques would impact brain connectivity in the same way. Nonetheless, there is an EEG study (Neuling et al., 2013) in which the participants' occipital cortex (anode: Oz) was stimulated in the individual alpha frequency (IAF) to compare the aftereffects of stimulating in two different conditions: eyes-closed and eyes-open. These authors found that alpha power only differs between tACS stimulation and sham in the eyes-open condition. The coherence between two parietal electrodes (P3-P4), however, was only increased after tACS stimulation with eyes-closed (not with eyes-open). Therefore, there was a difference in the aftereffects of stimulating the areas with eyes-open and eyes-closed, which support the idea that the effects of tCS are dependent on the brain state, and for tACS in particular, on the ongoing brain oscillations. Considering the time varying nature of the oscillatory brain activity, new real time protocols are being developed to adjust the stimulation frequency according to the brain oscillations in real time (Boyle and Frohlich, 2013).

## Creativity, brain stimulation, and network analysis

Creativity is a multidimensional construct that can be investigated from a number of different perspectives, from its associated processes such as convergent and divergent thinking (Sawyer, 2012), passing through the creative person, product and press or environment (Rhodes, 1961). In this paper, we borrowed the following definition of creativity from Plucker et al., 2004: *“Creativity is the interaction among aptitude, process, and environment by which an individual or group produces a perceptible product that is both novel and useful as defined within a social context” (p. 90)*. In the field of Cognitive Neuroscience, the main focus of creativity research is on the processes involved in the creative thinking, including divergent and convergent thinking (Luft and Bhattacharya, 2014). Divergent thinking refers to the capacity of generating novel and original ideas to open-ended problems (e.g., think of as many unusual uses of a brick). Convergent thinking, on the other hand, refers to the process of finding a correct solution to a closed-ended problem, such as a puzzle. In the real world, however, the creative process involves both divergent and convergent thinking (Sawyer, 2012).

There are only a few studies on how we can boost creativity using tCS (Cerruti and Schlaug, 2009; Chi and Snyder, 2011, 2012; Metuki et al., 2012; Chrysikou et al., 2013), which are described in Table 2. On the divergent thinking study (Chrysikou et al., 2013), the authors positioned the cathodal electrode over the left or the right prefrontal cortex and the anode over the mastoid, in an attempt to inhibit the left or right DLPFC. They found that cathodal stimulation over the left DLPFC, but not over the right DLPFC, was associated with quicker responses on the uncommon uses task (the participants were asked to generate common or uncommon uses for presented objects). The authors suggested that this result is coherent with the idea that divergent thinking depends on transient hypo-frontality, but in this case, it was found to be specific to the left hemisphere.

**Table 2:** **Studies on tCS and creativity**.

**Study**	**Creative process**	**Technique**	**Parameters/location**	**Duration**	**Design**	**Paradigm**	**Results**
Chi and Snyder, 2012	Insight	tDCS	1.6 mA (30 s raising), electrode 35 cm^2^ Anode: right ATL Cathode: left ATL	10 min	Within-participants: pre-during-post Between: active vs. sham	9-dot problem	40% of the active group were able to solve the problem; none of the sham group solved it
Chi and Snyder, 2011	Insight	tDCS	1.6 mA (30 s raising) electrode 35 cm^2^ Anode: right ATL Cathode: left ATL	10 min	Between participants: (1) L− R+; (2) L+ R−; (3) Sham stimulation	Matchstick problems	60% of the participants in the L-R+ group were able to solve the difficult problems whereas lower than 20% in the other groups solved it
Metuki et al., 2012	Insight	tDCS	1 mA (30 s raising) electrode 35 cm^2^ Unilateral active Anode: left DLPFC (F3); Cathode: right OFC (Fp2)	11 min (5 min pre + 6 online)	2 × 2Within-participants: (1) Active vs. sham (sessions separated by a week); (2) Easy vs. Hard	RAT (CRA) with limited time (to investigate solution identification rather than generation)	They found that stimulation did not affect the rate of solution for either hard or easy problems. However, they found an interaction between stimulation and difficulty for solution recognition, as the participants in the active stimulation group were more able to recognize correct solutions for hard problems
Cerruti and Schlaug, 2009	Insight	tDCS	1 mA Anode: 16.3 cm^2^ Cathode: 30 cm^2^ Experiment 1 Anode: left DLPFC Cathode: right OFC Experiment 2 Anode: left DLPFC Cathode: right DLPFC	20 min 16 min stimulation + 4 min stimulation during the verbal fluency task	Within-participants design with (3 h session): (1) Anodal electrode location: F3 vs. right supraorbital region (Experiment 1); F3 vs. F4 (Experiment 2); (2) Condition: active anodal, active cathodal, sham	Verbal Fluency (VF) + RAT (CRA) with 30 s to solve	They found that the stimulation did not improve VF, but was associated with higher solution rates when the stimulated area was above the left DLPFC. The two experiments showed the same result, with higher solution rates for anodal on the left DLPFC
Chrysikou et al., 2013	Divergent thinking (flexible tool use)	tDCS	1.5 mA 25 cm^2^ electrodes Cathode: F7 or F8 Anode: on the contralateral mastoid (the main purpose was to cause inhibition of PFC)	20 min (including 10 s ramp-up + 10 s ramp down). Stimulation began for 180 s prior to the tasks	Between-subjects design with two factors: Stimulation protocol (groups): (1) Cathodal Left (F7) and anodal on mastoid; (2) Cathodal Right (F8) and anodal on mastoid; (3) Sham Task (groups): (1) Common uses; (2) Uncommon uses	Div. Thinking: participants were asked to generate either (1) common vs. (2) uncommon uses for the objects presented on the screen (60 grayscale pictures). Each participant was assigned to only one of these two conditions. The performance was measure to response onset time	There was a significant interaction between stimulation protocol and task condition, since cathodal over the left PFC was associated with in an decrease in the response times for the uncommon uses task. There was no difference in performance between stimulation conditions in the common uses task. In addition, cathodal stimulation over the left PFC was associated with lower number of response omissions in the uncommon uses task only (no difference in the common uses task)

Most of the studies cited in Table 2 investigated creative insight, which is a convergent thinking process. In one of the insight studies (Cerruti and Schlaug, 2009) it was found that anodal stimulation over the left DLPFC increases the solution rate in a convergent thinking task, the “Remote Associate Task—RAT.” In this task, the participants have to find a fourth word which makes a compound word with three words presented on the screen (e.g., food/forward/break; solution: fast). However, another study (Metuki et al., 2012) observed that anodal stimulation over the left DLPFC did not improve the solution rate when subjects were given less time to solve the problem (7 s compared to 30 s), but it did improve subjects' ability to recognize correct solutions to hard RAT problems. The authors suggested that the left DLPFC is involved in recognizing the correct solution rather than generating it, a role that has been attributed to the right hemisphere (Bowden and Jung-Beeman, 2003), especially the right anterior area (Jung-Beeman et al., 2004). A slightly different account can be suggested based on two experiments (Anderson et al., 2009) monitoring the subjects brain responses (fMRI) while they solved a compound-word RAT task (Experiment 1) and another similar paradigm which allowed faster responses (Experiment 2). They observed that while the LIPFC was associated with the word search in memory or memory retrieval, the ACC was associated with the processing of solutions. In both experiments, the LIPFC activity increased when the participants were trying to find a solution, but from the moment they reached it, the ACC activity increased and the LIPFC returned to baseline levels.

The other two insight studies (Chi and Snyder, 2011, 2012) focused on other insight problems that are less dependent on verbal processes (matchstick and 9-dot problems). In both studies (Chi and Snyder, 2011, 2012), the stimulation protocol positioned the cathodal electrode over the left anterior temporal lobe (ATL), and the anodal over the right ATL. The authors found that this protocol increased the solution rates to these problems in relation to sham stimulation (between groups).

Therefore, for insight studies, it seems that the left DLPFC, and the right ATL play a role in finding solutions for convergent thinking. Nonetheless, little is still known about how these areas communicate to generate a solution as none of these studies combined tCS with any neuroimaging techniques, making it difficult to know whether these changes in performance are indeed caused by the excited/inhibited areas (Chi and Snyder, 2011, 2012; Chrysikou et al., 2013), by a connection between the stimulated/inhibited area, or by a compensatory network mechanisms that may be triggered by inhibiting those (as in finding 4 described in the previous section). In addition, in the human motor system evidence that bilateral tDCS evokes a suppression of one hemisphere but a facilitation of the other is somewhat mixed (see Nitsche and Paulus, 2000; Mordillo-Mateos et al., 2012; Hasan et al., 2013). Thus, the biological impact of bilateral tDCS (and by virtue the mechanisms that modulate previously reported enhancements in creativity following bilateral tDCS—e.g., Chi and Snyder, 2011, 2012) remains unclear.

Previous studies on the brain activity underlying creativity found, for example, that divergent thinking is associated with higher functional connectivity between medial prefrontal cortex (mPFC) and posterior cingulate cortex (PCC), both of which are key nodes of the DMN (Takeuchi et al., 2012). It could be that inhibiting the lateral prefrontal cortex may result in higher activation of the medial prefrontal, but this can only be tested by combining tCS with neuroimaging. There are a number of studies suggesting that creativity is associated with higher connectivity, both structural (Jung et al., 2010a,b; Takeuchi et al., 2010) and functional during rest (Jaušovec and Jaušovec, 2000a; Kounios et al., 2008; Takeuchi et al., 2012) and task alike (Jaušovec and Jaušovec, 2000b; Bhattacharya and Petsche, 2002, 2005; Razumnikova and Larina, 2005). Further, there are recent suggestions that creative ideas may reside in dynamic activation patterns of spontaneous brain networks (Wiggins and Bhattacharya, 2014). On account of these works, we believe that an important step towards improving creativity through tCS would be to combine brain stimulation with neuroimaging and advanced analysis techniques, in order to find how the brain networks mediate the improvements in creative processes observed in the cited studies. Importantly, techniques such as tACS allow targeting both long range and local synchronization (Ali et al., 2013).

**Figure 2:**
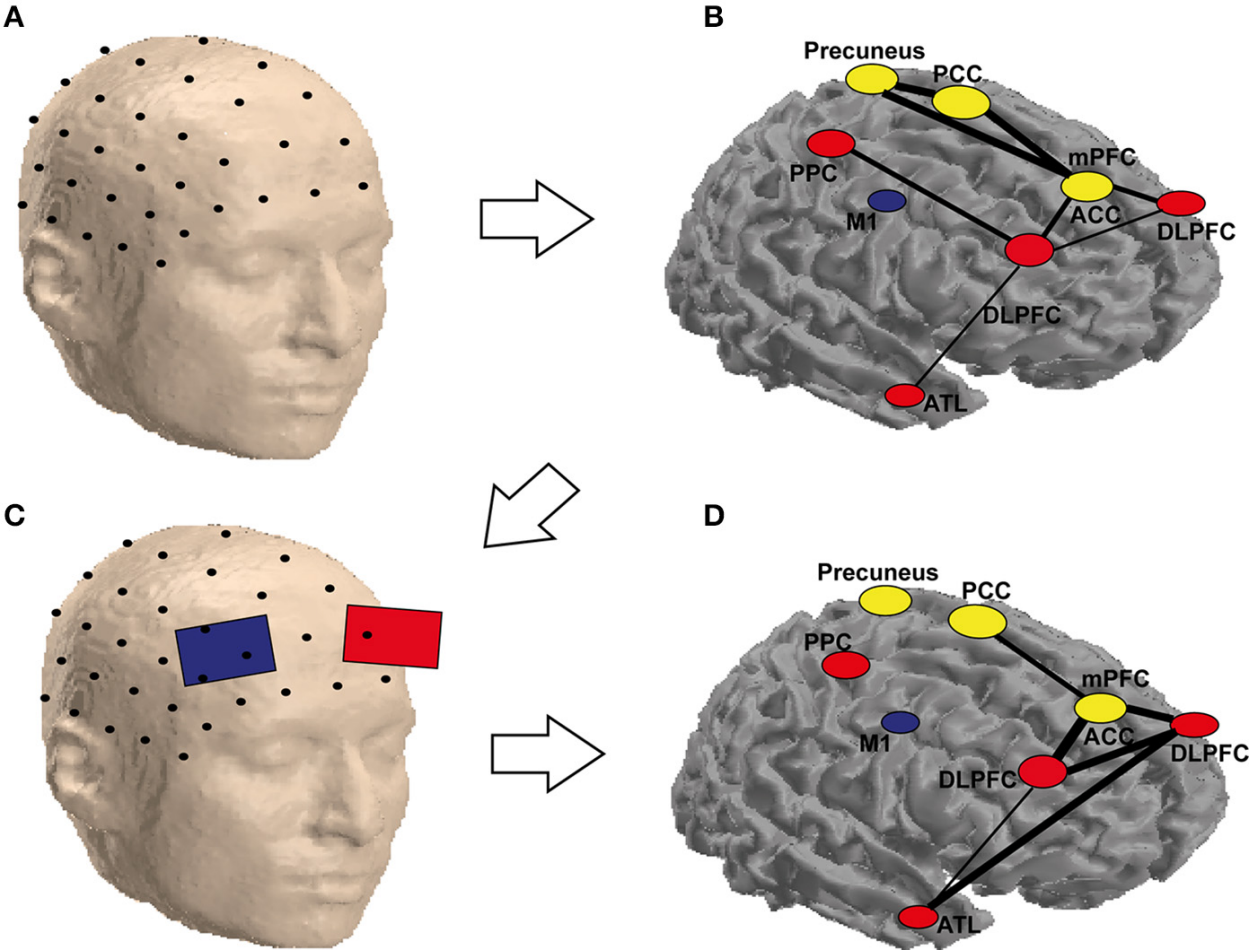
**Schematic representation of the possible combination of tCS and functional (EEG) brain connectivity to enhance creativity**. An array of scalp electrodes, a subset of which is depicted in **(A)**, is recorded. In **(B)** is possible to identify ROIs which are highly connected (yellow) and the ones that are flexible hubs (red) or less connected areas (blue) during a solution vs. non-solution RAT task. This helps identifying the target electrodes to stimulate as in **(C)**. Stimulating these electrodes may not only eliminate the differences for the corresponding nodes, but also reduce them for areas in the same network, which are not stimulated but functionally connected to the targeted ones as depicted in **(D)**. It is worth noting that the labels and the networks drawn in the figure are only for demonstration, as they are not precisely equivalent to their anatomical locations (they are only approximate locations on a surface, the areas in yellow are located in the medial area of the brain, which cannot be seen in a cortical mesh). The areas are abbreviated as follows: PCC, posterior cingulate cortex; PPC, posterior parietal cortex; M1, primary motor cortex; mPFC, medial prefrontal cortex; ACC, anterior cingulate cortex; DLPFC, dorsolateral prefrontal cortex; ATL, anterior temporal lobe.

Based on the previous discussions, we propose a hypothetical approach for combining neuroimaging, connectivity and brain stimulation for improving creativity (Figure 2). In this approach, the research starts with the graph theoretical analysis of the connectivity between the regions involved in a certain process (e.g., convergent thinking—insight) as in Figure 2A. Using the discovered cognitive connections (Figure 2B), a protocol for stimulation is determined (Figure 2C). In Figure 2A, we showed a couple of regions involved in creativity. Immediately after the stimulation, the connectivity patterns (in identical conditions of the analyzed patterns in Figures 2A,B) are analyzed against control stimulation protocol, e.g., sham stimulation. The results are presented as a map, with the edges linking the nodes representing here the strength of the difference in connectivity between active and sham stimulation (or any other control or contrast of the experiment), as in Figure 2D. In Figure 2 hypothetical example, the connectivity changed after stimulation of the left DLPFC during a RAT paradigm, especially among the temporal and frontal areas (Figure 2D). In the hypothetical example, there was an increase in communication between ATL and DLPFC and between the DLPFC and the ACC, which could represent coordinated (DLPFC) search of the solution in memory (ATL) and the recognition of the correct solution (ACC), from DLPFC to ACC. Note that this approach does not account for the direction of the interactions, which could be also tested using effective connectivity analysis techniques such as dynamic causal modeling or Granger causality. In divergent thinking, the approach would probably result in a higher change over the posterior rather than anterior regions.

## Limitations of graph-theory and brain stimulation

Notwithstanding the advances emerging from the combination of tCS and neuroimaging for the understanding of the connectivity changes in response to brain stimulation, there are many limitations of both methods which can be thought as new challenges for this enterprise. First, despite its usefulness in characterizing brain network during cognitive functions (Sporns, 2014), the application of graph theory is not problem-free (Fornito et al., 2013). The approach, after all, is based on sophisticated mathematical techniques that require judicious choices at various steps of the analysis. Perhaps the most obvious one is the need to choose among a number of possible strategies to reconstruct the networks, which do not always lead to a convergent or consistent outcome. For instance, one has to decide whether the links will be weighted or unweighted, directed or not, whether to use a fixed value or a fixed link density across participants as a threshold or to use statistical controls such as surrogate data analysis in order to determine the significance in a per-link basis. Further, especially in the case of EEG/MEG, many different bivariate indices of functional/effective connectivity are available (e.g., Niso et al., 2013). Besides, spurious characterization of a network could result from an inappropriate temporal and/or spatial sampling of the underlying systems (Bialonski et al., 2010, 2011). Last, but not least, the relationship between connectivity at the sensor/electrode level and connectivity at the neural source level is more complicated than traditionally assumed (Ewald et al., 2012). Nevertheless, we argue that graph theory is a very useful tool to study the synchronized activity between different brain areas underlying most cognitive functions, as it can be used to characterize these patterns of brain connectivity. Moreover, it can be also used to understand how local changes (whether internally or externally generated) are able to affect other distant brain areas. For instance, in focal epilepsy research a paradigm shift is currently taking place whereby attention is increasingly not toward the epileptic focus itself, the classical and obvious target area of the neuroscientific research on epilepsy, but the epileptic network and its characteristics (Lehnertz et al., 2014), offering a tremendous potential in minimizing the extent of surgical intervention.

The tCS methods also face limitations that have to be considered when attempting to develop protocols to boost specific cognitive functions. Currently, there are many issues related to our lack of understanding of how tCS, in its different modalities, shapes neural activity. First, as tCS methods in humans are non-invasive, the stimulation is applied to the skull rather than directly to the brain, meaning that the spatial resolution of tCS is diffuse due to skull dispersion. The spatial resolution can be improved with small stimulation electrodes (Datta et al., 2009), but it still does not overcome the problem that the stimulation is indirect and the electrodes often too large for such focal stimulation of a small cortical area. The second limitation, which is closely related to the first, is the lack of control of the stimulation current reaching the brain as the current is altered as it passes through the skull to be then conducted by the cerebrospinal fluid to the brain. Individual differences in skull thickness and shape may interact with how much current is actually reaching the brain (Datta et al., 2009). Therefore, even with advanced modeling of the current (Wagner et al., 2007; Shahid et al., 2014), it is difficult to predict how much current is actually reaching the brain. Third, the stimulation effects, as stated earlier in the Introduction, are not straightforward. For example, anodal stimulation does not always cause increase excitability, and vice versa for cathodal (Nitsche and Paulus, 2000; Antal et al., 2004; Moliadze et al., 2010). Further, other factors such as increasing the intensity (Batsikadze et al., 2013) or including a task during stimulation (Antal et al., 2007) can also change the effects in a complicated fashion. Therefore, it is important for these factors to be controlled carefully in tCS studies. Moreover, when one considers these limitations in conjunction with the fact that stimulating one area of the brain can affect a network of regions, it hampers the possibility of claiming “cause-effects” relations between a single stimulated brain region and specific cognitive functions as the stimulation effects are not entirely known.

## Conclusion

In this paper, we discussed the possibility of combining tCS with neuroimaging and graph theory for analysing the impact of brain stimulation on brain connectivity. In doing so, we highlight how graph theoretical analysis can help in understanding how the brain networks are affected by tCS in specific locations. In addition, we suggest that the knowledge of structural connectivity pathways can be used to target a network of brain areas rather than a single area and that ongoing brain connectivity during and just after (during the aftereffects period) tCS is an important factor in determining the connectivity changes in response to stimulation. In the future, we suggest using graph theory not only to understand the network effects of stimulating different brain areas, but also to develop tCS protocols that can target connectivity rather than individual brain areas.

### Conflict of interest statement

The authors declare that the research was conducted in the absence of any commercial or financial relationships that could be construed as a potential conflict of interest.
